# Prosthodontics Using Removable Platform Switching Technologies (Multiunit, On1) as Exemplified by Conical Connection Implant Systems for Early and Immediate Loading

**DOI:** 10.1155/2021/6633804

**Published:** 2021-04-19

**Authors:** Roman Studenikin

**Affiliations:** Dental Clinic, Vash Stomatolog, Bulvar Pionerov, Voronezh 394038, Russia

## Abstract

The removable platform switching technology (multiunit, Оn1) was tested intraoperatively using the passive placement technique as exemplified by a conical connection implant system, which makes it possible to visually control the placement of these platforms with respect to the alveolar bone in the correct orthopedic position. The technology is characterized by a rapid epithelialization of tissues around the base platform until the final integration of the implant, minimal trauma in the emergence profile zone, and an improved minimally invasive orthopedic protocol for working on a removable platform switching base.

## 1. Introduction

Conical connection implants are gaining popularity since their use with a specific surgical protocol allows the early loading due to the implant macrodesign by, for example, placing the implant subcrestally, immersing it below the level of the alveolar ridge apex by 2-3 mm [1–3]. Having reached the primary stability of at least 30–45 N/cm^2^ during the implant placement, it becomes possible to proceed to the stage of prosthetics immediately or after 6–8 weeks, which makes it possible to quickly conduct orthopedic rehabilitation in comparison with the well-known implantation methods [4, 5].

A classical two-stage implantation using implant systems with a conical connection is accompanied by suturing the implant under the periosteum for 3–6 months. This often leads to additional unpredictable bone growth around the implant neck and plug [6–8]. At the stage of uncovering the implant, the overhanging part of the bone tissue over the implant plug is removed, and the soft tissue plastic surgery and the gingiva former placement are carried out, which additionally causes injury to the alveolar bone. After the final integration, it becomes possible to install an impression coping and fabricate a provisional crown, forming the emergence profile depending on the anatomy of the future permanent prosthesis [9–11].

**Figure 1:**
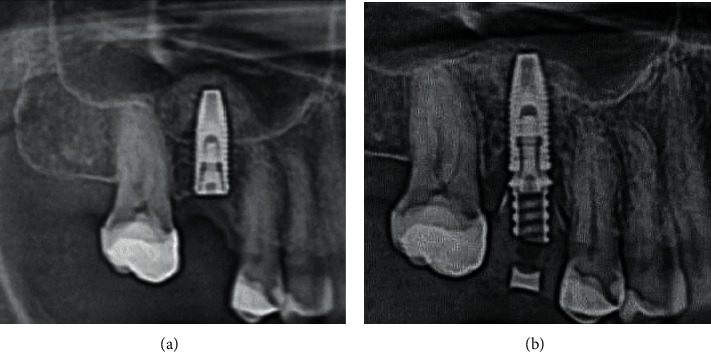
Dynamics of bone resorption: (a) complete implant integration after 6 months; (b) provisional crown on the implant 3 weeks after placement.

Numerous manipulations from the moment of uncovering the implant to the final prosthetics lead to additional microtrauma of the bone area around the implant and the emergence profile [12, 13]. In some places, the provisional prosthesis puts uncontrolled pressure on the peri-implant tissues, which causes rupture of hemidesmosomal junctions [14]. As a result, unpredictable bone resorption around the implant neck often occurs already at the stage of provisional prosthetics, within a month after the implant integration (Figure 1).

The problem of bone tissue loss around the implant is eliminated by applying various customized prosthetic components made of biocompatible materials (zirconium dioxide abutment and customized PEEK former) and installing them intraoperatively [15–19].

The disadvantages of these techniques are economic and temporal factors, as well as the lack of control of the emergence profile interface on the abutment after implant integration [20, 21].

**Figure 2:**
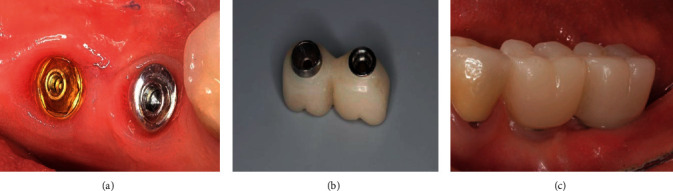
Temporary rehabilitation of a patient from the level of removable platform switching bases at the stage of prosthetics.

To eliminate these shortcomings, it became necessary to place special removable prosthetic components on the implant with platform switching directly during surgery, having previously carried out a preparatory stage for reducing the alveolar bone around the implant. Having installed the prosthetic component intraoperatively, it becomes possible to carry out all manipulations only from the level of this base (Figure 2).

Platform switching technique (PLS) is used as part of prosthetic components on various conical or flat connection implant systems [22]. The titanium base is a prosthetic component with a smaller diameter than that of the implant platform. The platform switching height varies from 1 to 4 mm.

The placement of a single prosthetic component throughout the entire treatment stage, including surgery, makes it possible to visually control their passive placement in the correct orthopedic position and prevent the loss of the alveolar bone around the implant.

The aim of the work is the application of removable switching platform technology intraoperatively as exemplified by multiunit On1 systems using the developed passive fixation technique followed by subsequent early or immediate loading with the dental prosthesis.

## 2. Materials and Methods

### 2.1. The Application of Removable Platform Switching Technology

The On1 titanium base from Nobel Biocare coated with biocompatible material is a platform switching structure that turns into a base with a separate screw for attaching to the implant. The manufacturer delivers the product in sterile packaging.

**Figure 3:**
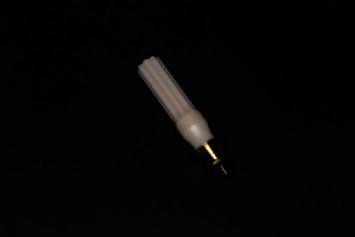
Removable On1 platform switching base.

The On1 base has a disposable plastic holder that holds the attachment screw and serves as a screwdriver when it is inserted into the implant platform. The screw head has an internal thread for attaching a titanium abutment to support the future prosthesis (Figure 3).

The On1 base (*d* = 4.8 mm) has a platform switching height of 1.75 mm and 2.5 mm for various implant diameters. The seat is provided for the titanium universal abutment, both for single-piece prosthesis with an antirotation grip and without it for bridges.

The torque force on the screw when installing the base into the implant should be 30–35 N/cm^2^, but not more than the initial implant stability during its placement.

### 2.2. The Technology of Intraoperative Base Placement

Using the special NobelClinician software and computed tomography, the future orthopedic positioning of the implant is planned; the implant's diameter and length are selected with respect to the bone volume, as well as the immersion depth. The level of implant primary stability is preliminarily determined by the bone type; the gingival biotype and gingiva height are assessed. These parameters determine the optimal biological width of the soft tissue around the implant for future prosthetic aesthetics and implant survival.

**Figure 4:**
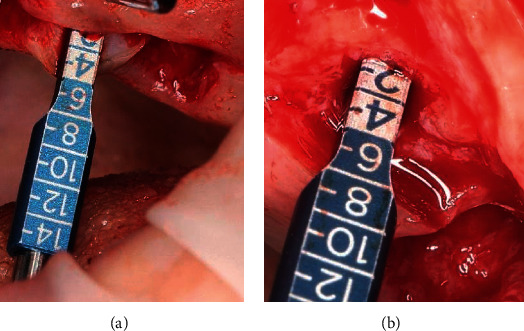
Control of the reduction depth: (a) measurement of the thickness of the attached mucosa; (b) measurement of the reduction depth of the alveolar ridge.

During the surgery, the mucoperiosteal flap is folded back, and with the help of a straight increasing tip and a spherical bur with a diamond coating, a groove-like reduction of the alveolar bone in the zone of future implant placement is performed. The reduction diameter should be larger than the diameter of the implant to be placed by 2 mm. The reduction depth is controlled in relation to the soft tissue thickness and future On1 base height as follows (Figure 4):

  By the biological width (not less than 3.5–4 mm from the edge of the epithelium to the implant platform)
  By the thickness of the attached keratinized mucosa

**Figure 5:**
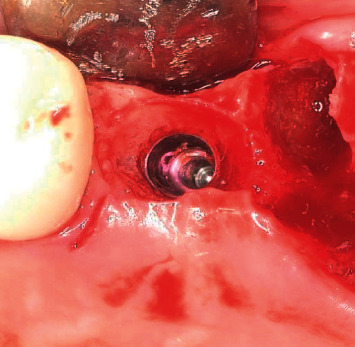
Preliminary bone reduction with implant placement.

The missing depth is obtained by bone reduction. After the implant placement with the desired immersion depth, the implant shaft is irrigated with an antiseptic solution (Figure 5).

**Figure 6:**
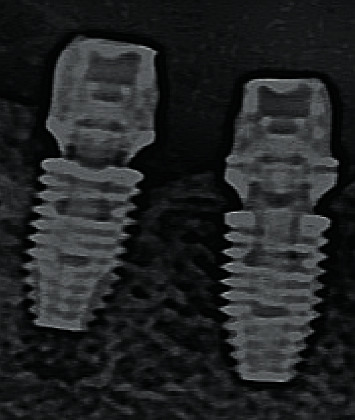
Radiographic control of the fit of the On1 base into the implant during surgery.

The On1 base is inserted into the implant using the holder. The platform switching height and the passivity of the base installation into the implant are visually controlled. The zone of the alveolar bone should not touch the lower part of the On1 base. Control of the fit of the base into the implant is performed radiographically (Figure 6).

**Figure 7:**
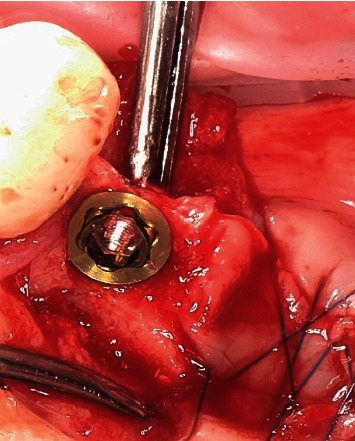
Placement of the removable On1 base into the implant.

With sufficient accuracy of the base fit to the implant platform, the torque force on the screw is 30–35 N/cm^2^, but not more than the implant primary stability (Figure 7).

Then, a special gingiva former is placed on the On1 base, and sutures are applied. Upon reaching the final integration, the gingiva former is removed, the emergence profile is assessed, and an early loading on the implant is performed with a provisional prosthesis fabricated in a dental laboratory. Before the final fixation of the prosthesis, it is necessary to check the torque force on the removable On1 base, which should be 35 N/cm^2^.

For the On1 removable base, there is a variety of abutments: for provisional prostheses, for permanent screw-retained prostheses in the form of a universal abutment, an aesthetic abutment for cement-retained prostheses, as well as for bridges.

The intraoperative placement of a sterile removable platform switching On1 base leads to mechanical sealing and, as a result, to the absence of bacterial flora in the implant shaft, providing a barrier for the alveolar bone as well as protecting soft tissues through the entire stage of implant integration and preserving the emergence profile morphology until the beginning of orthopedic treatment.

### 2.3. Application of Removable Straight and Angled Platform Switching Bases as Exemplified by Multiunit Systems

In various clinical situations, it becomes necessary to place the implant at an angle of 0 to 30° to achieve the greatest primary stability in the bone tissue for immediate loading, as well as to bypass important anatomical formations.

The bases with a system for changing the platform angle are used in prosthetic treatment of patients with complete and partial loss of teeth, when bridges are placed.

**Figure 8:**
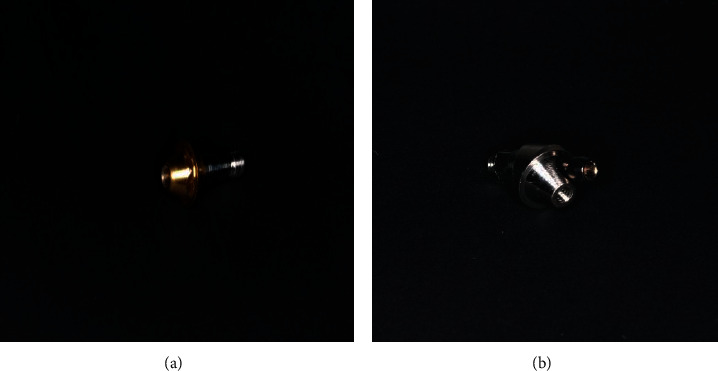
Straight and angled multiunit systems.

The removable multiunit titanium base has a single platform that turns into a screw. The base contains a holder for easy insertion into the implant. The platform of the removable base has an internal thread for attaching prosthetic components. Nobel Biocare manufactures straight and angled (17 and 30°) multiunit systems. The latter have an additional screw to change the inclination angle of the platform. Platform switching heights vary from 2.5 to 4.5 mm at the conical implant connection. The product is delivered in sterile packaging (Figure 8).

**Figure 9:**
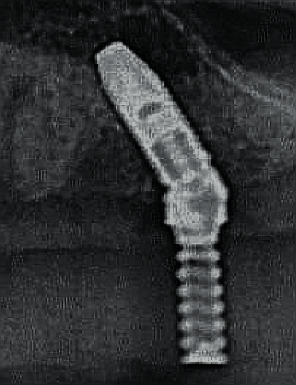
Radiographic control of the angled multiunit base fit into the implant.

After planning the implant placement in the correct orthopedic position, the horizontal and vertical reduction is performed in the zone of the future implant in the form of a groove-shaped depression. The implant is installed with the platform immersed below the level of the alveolar ridge, taking into account the gingival biotype. The implant shaft is irrigated, and the multiunit base is installed with specific height and inclination angle. The fit is controlled radiographically (Figure 9).

The base should not put pressure on the alveolar bone. The torque force is set with a special torque key to 30 N/cm^2^ for a straight and 15 N/cm^2^ for an angled multiunit system, respectively. After placing the removable platform switching bases, a provisional prosthesis is installed. During the period of final implant integration and replacement of the provisional prosthesis with a permanent one, the torque force is additionally monitored on a multiunit base.

**Figure 10:**
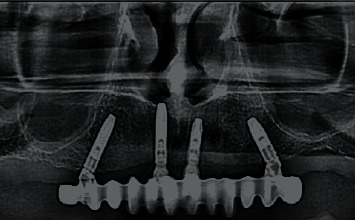
Control of the fit of a titanium bar of a permanent screw-retained multiunit-supported prosthesis.

Sampling of the alveolar bone, passive placement of the bases into the implant, and the absence of compression in this critical zone lead to directed vertical bone growth to the height of the platform switching of the removable titanium base, which is stable for a long time. Besides, the placement of removable platform switching bases makes it possible not only to obtain a predicted directed bone tissue volume but also allows one to work in the correct orthopedic position with implants and achieve the accuracy of the fit of the future prosthesis (Figure 10).

### 2.4. For Conventional Technology

After the implant placement with a primary stability of at least 25 N/cm^2^, a gingiva former is used to reduce the stage of orthopedic treatment, which allows quick access to the implant at the stage of prosthetics.

**Figure 11:**
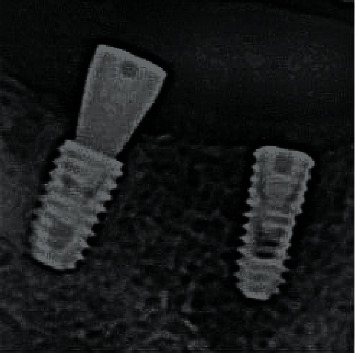
Subcrestal implant placement, intraoperative placement of the gingiva former.

As shown on the X-ray image after 6 weeks, the implant is placed below the level of the alveolar bone (Figure 11).

**Figure 12:**
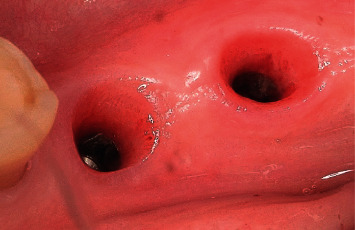
Emergence profile after gingiva former, 8 weeks after implant placement.

Such immersion of the implant into the bone results in a deep emergence profile (Figure 12).

At the stage of prosthetics, the gingiva former is unscrewed, an impression transfer is installed, and impressions are taken to make a provisional crown in a dental laboratory. The emergence profile is scanned on the plaster model together with the provisional abutment. The platform switching height for provisional abutments is standardized by the manufacturer at 1 and 3 mm and is usually adjusted by machining depending on the depth of the emergence profile.

**Figure 13:**
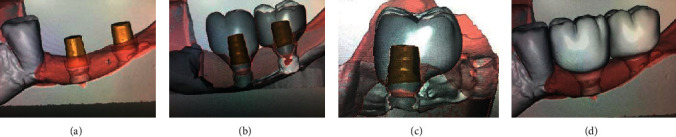
Stages of modeling temporary crowns on implants in the program.

Then, in a special program, the image of the future provisional prosthesis is modeled with a smooth transition from the given height of the titanium provisional abutment to the neck of the future crown (Figure 13).

The gingiva former is removed, and a provisional crown is placed on the implant with a torque force of 25 N/cm^2^.

**Figure 14:**
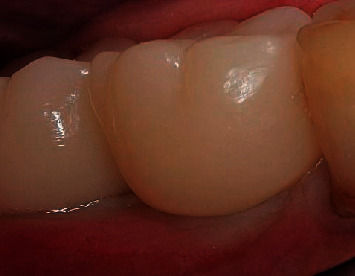
Soft tissue ischemia during the placement of the provisional prosthesis.

The crown placement, given the small diameter of the gingiva former, is always accompanied by soft tissue ischemia, despite the use of a provisional platform-switching abutment (Figure 14).

**Figure 15:**
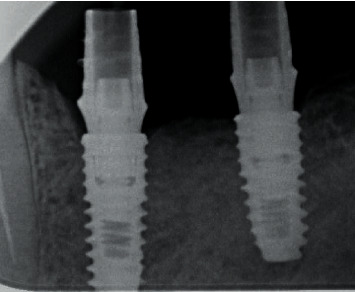
Bone compression during the placement of the provisional platform switching system on the implant.

The deep layers of the epithelium often come into contact with the bone structures of the alveolar bone overhanging the implant, located at different levels during subcrestal implant placement (Figure 15).

Despite visual and X-ray control during the placement of the provisional platform switching system, a certain compression of the bone tissue cannot be completely avoided. This often leads to uncontrolled bone remodeling in this area.

**Figure 16:**
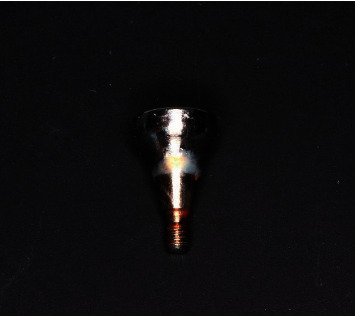
Contamination of the gingiva former.

The disadvantages of this technique are the frequent removal and placement of a gingiva former with a contaminated surface (Figure 16).

**Figure 17:**
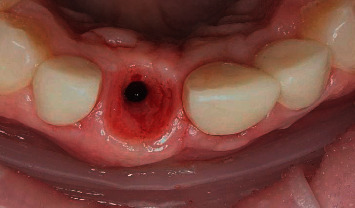
Microruptures in deep layers of the epithelium.

The subsequent frequent removal of the provisional crown for induration of the interdental contact points and the correction of the volume and shape of the prosthesis leads to frequent rupture and healing of the epithelium during the entire stage of temporary rehabilitation, which can last up to a month (Figure 17).

## 3. Results

In the main group, 15 patients underwent a dental implantation surgery with primary stability from 30 to 40 N/cm^2^. Removable platform switching bases were intraoperatively installed on the implants. 10 patients in the experimental group underwent delayed implantation followed by early loading with a prosthesis.

**Figure 18:**
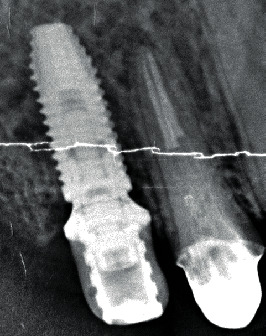
Checking the fit of the On1 base into the implant on the day of surgery.

In the entire group, X-ray images were made at the time of surgery to check the accuracy of the placement of the superstructure with respect to the implant platform (Figure 18).

**Figure 19:**
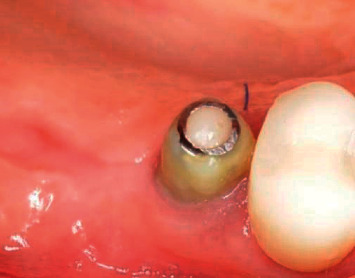
Condition of the soft tissues after suture removal on the 10th day.

On the 10th day after suture removal, there were no soft tissue inflammations (Figure 19).

**Figure 20:**
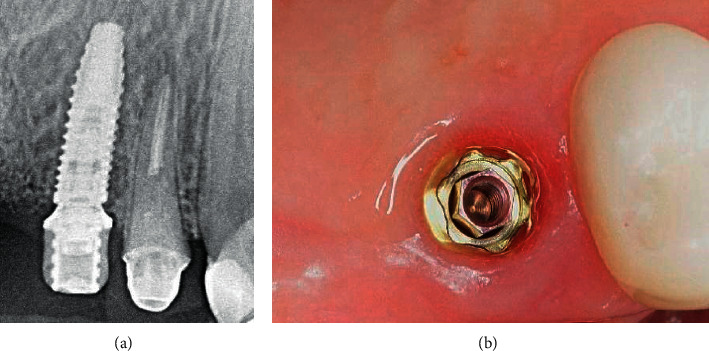
(a) Bone formation under the On1 base (8 weeks). (b) Condition of the soft tissues around the On1 base (8 weeks).

After 6–8 weeks, radiographic control of the implant integration and assessment of the emergence profile were performed (Figure 20).

**Figure 21:**
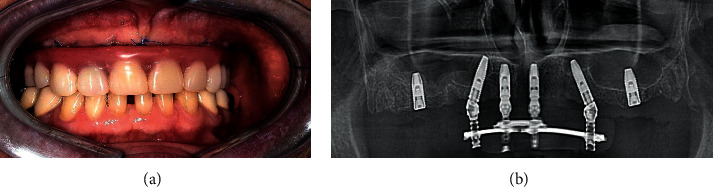
Immediate loading on the day of surgery using multiunit system and radiographic control.

There is a vertical growth of the alveolar bone from the implant to the base. The rest of the patients in the main group (5 people) underwent implantation with immediate loading with the prosthesis on the day of surgery (Figure 21).

**Figure 22:**
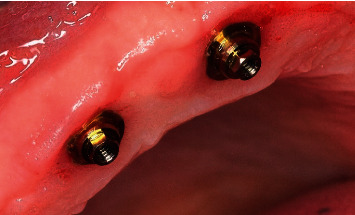
Formation of the emergence profile around the removable multiunit base.

At the examination stage, on the 10th day, the wound healing occurred by primary intention, and the sutures were good. Epithelialization of soft tissues was complete, and no inflammation was detected (Figure 22).

**Figure 23:**
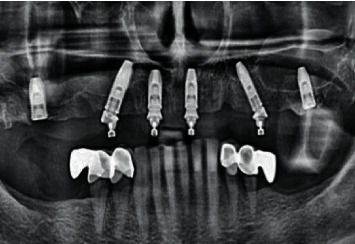
Radiographic control of implant integration (8 weeks).

X-ray images taken to check the integration after 6 weeks showed bone matrix formation around the removable multiunit base (Figure 23).

In the comparison group, which consisted of 10 patients, a dental implantation surgery was performed in the lower jaw area and gingiva formers of various diameters and heights were installed intraoperatively. The implant primary stability varied in the range from 30 to 35 N/cm^2^.

All patients underwent early loading after 8–12 weeks with provisional crowns fabricated in the dental laboratory to shape the emergence profile before prosthetics with a permanent prosthesis.

**Figure 24:**
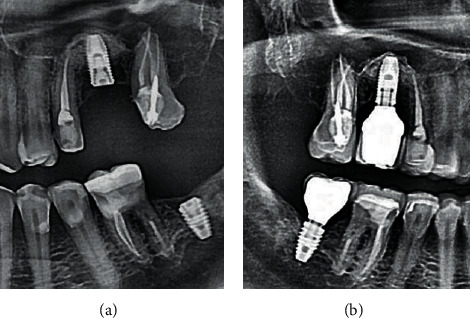
X-ray control: (a) implant integration after 16 weeks; (b) bone remodeling around the implant neck 4 weeks after placement of the platform switching prosthesis.

X-ray images 4 weeks after the placement of the provisional prosthesis showed bone remodeling around the implant in two out of ten patients in this group (Figure 24).

The data presented demonstrate that the lack of implant hermeticity, the frequent placement of various prosthetic components throughout the patient's rehabilitation period, and the decontaminated surface of the gingiva former lead to unpredictable bone loss around the implant at the stage of emergence profile formation. It is not possible to achieve 100% success in the stability of soft tissues and bone around the implant after their application.

## 4. Conclusions

The application of this technique yields the following:

  Predictable growth of the alveolar bone to the height of the removable platform switching base
  Rapid epithelization of tissues around the base platform already before the final implant integration
  The possibility of performing all orthopedic manipulations from the level of the removable switching platform base at all stages of treatment, minimally traumatizing the emergence profile zone, which ultimately leads to a spontaneous predictable formation of the biological width
  Improved minimally invasive orthopedic protocol on a removable platform switching base

Consequently, the removable platform switching technology makes it possible to achieve a stable aesthetic result in the shortest possible time.

## Data Availability

Access to data is restricted. The data can be made available upon request to studenikin@yahoo.com.
